# Human Umbilical Cord-Derived Mesenchymal Stem Cell Therapy Ameliorates Nonalcoholic Fatty Liver Disease in Obese Type 2 Diabetic Mice

**DOI:** 10.1155/2019/8628027

**Published:** 2019-11-03

**Authors:** Bing Li, Yu Cheng, Songyan Yu, Li Zang, Yaqi Yin, Jiejie Liu, Lin Zhang, Yiming Mu

**Affiliations:** ^1^Department of Endocrinology, The First Medical Center of PLA General Hospital, Beijing 100853, China; ^2^Outpatient Department, The Fifth Medical Center of PLA General Hospital, Beijing 100039, China; ^3^Department of Molecular Biology, Institute of Basic Medicine, School of Life Science, PLA General Hospital, Beijing 100853, China

## Abstract

Nonalcoholic fatty liver disease (NAFLD) is increasingly common among patients with type 2 diabetes mellitus (T2DM). The two conditions can act synergistically to produce adverse outcomes. However, the therapeutic options for patients with NAFLD and T2DM are currently limited. Human umbilical cord-derived mesenchymal stem cells (UC-MSCs) have shown therapeutic potential for diabetes and hepatic disorders such as liver cirrhosis and fulminant hepatic failure. The present study is aimed at investigating the effect of human UC-MSCs on a mouse model of NAFLD and T2DM, characterized by obesity-induced hyperglycaemia, dyslipidaemia, hepatic steatosis, and liver dysfunction. Thirty-week-old male C57BL/6 db/db mice were infused with human UC-MSCs or phosphate-buffered saline (PBS) via the tail vein once a week for six weeks. Age-matched male C57BL/6 wild-type db/+ mice were used as controls. Body weight and random blood glucose were measured every week. One week after the sixth infusion, intraperitoneal glucose tolerance tests and insulin tolerance tests were performed and the blood and liver were harvested for biochemical and histopathological examinations. Quantitative real-time reverse transcriptase polymerase chain reaction (qRT-PCR), immunofluorescence staining, and western blot were performed to monitor the expression of the lipid metabolism- and regulatory pathway-related genes. UC-MSC infusions significantly ameliorated hyperglycaemia, attenuated the elevation of hepatic transaminases, and decreased lipid contents, including triglyceride, total cholesterol, and low-density lipoprotein cholesterol. Moreover, histological lesions in the liver diminished markedly, as evidenced by reduced lipid accumulation and attenuated hepatic steatosis. Mechanistically, UC-MSCs were found to regulate lipid metabolism by increasing the expression of fatty acid oxidation-related genes and inhibiting the expression of lipogenesis-related genes, which were associated with the upregulation of the HNF4*α*-CES2 pathway. Our results demonstrate that human UC-MSCs can ameliorate NAFLD and reverse metabolic syndrome in db/db mice. Thus, UC-MSCs may serve as a novel therapeutic agent for T2DM patients with NAFLD.

## 1. Introduction

With the changes in diet and lifestyle, obesity has become an alarming health crisis of the 21st century. Global estimates indicate that about 500 million people have obesity and are at risk of significant obesity-related morbidity [1]. Type 2 diabetes mellitus (T2DM) and nonalcoholic fatty liver disease (NAFLD) are both obesity-associated metabolic diseases, and they often coexist. The prevalence of NAFLD among T2DM patients is nearly 70%, which is at least 2-fold higher than that in the general population [2, 3]. On one hand, in T2DM patients with NAFLD, extra lipid accumulation in the liver leads to hepatocyte dysfunction, which adversely affects the glycaemic control and increases the likelihood of developing diabetic complications. On the other hand, the presence of T2DM worsens the course of NAFLD and augments the risk of developing cirrhosis and hepatocellular carcinoma. These interactions make the management of NAFLD and T2DM more complicated and challenging [4, 5]. Moreover, prognosis for patients with concomitant NAFLD and T2DM is worsened on account of increased risk for a life-threatening sequel such as cardiovascular disease and hepatocellular carcinoma, highlighting the need for improved treatment options [6, 7].

Mesenchymal stem cells (MSCs) are a heterogeneous subset of stromal stem cells that can be isolated from various adult tissues, such as bone marrow, mobilized peripheral blood, umbilical cord, and adipose tissue. They are superior to embryonic stem cells in terms of ethical concerns and immunogenicity [8]. MSCs harbour self-renewal capacity, multilineage differentiation potential, paracrine effects, and immunomodulatory properties, making them a promising tool in regenerative medicine. Human umbilical cord-derived mesenchymal stem cells (UC-MSCs) possess a unique combination of prenatal and postnatal stem cell properties and have higher proliferative potential and lower immunogenicity with allogeneic sources than other commonly used MSCs such as bone marrow-derived MSCs (BM-MSCs). Moreover, the noninvasive procurement and painless collection procedures make UC-MSCs a versatile candidate for clinical application [9, 10]. In liver diseases, UC-MSCs have been demonstrated to exert therapeutic effects on decompensated liver cirrhosis [11, 12], liver failure [13], chemical-induced liver injury [14, 15], and autoimmune liver diseases such as primary biliary cholangitis [16]. Meanwhile, UC-MSCs have been confirmed to alleviate insulin resistance and improve glucose homeostasis in high-fat diet/streptozotocin- (STZ-) induced T2DM rodent models by inducing phenotypic transition of macrophages, which is a manifestation of their immunomodulatory property [17, 18]. However, the therapeutic effect of UC-MSCs on NAFLD combined with T2DM has not been investigated. In this study, we evaluated the effect of UC-MSCs on NAFLD in db/db mice that spontaneously develop hyperphagia-induced obesity, T2DM, and NAFLD due to dysfunction of the leptin receptor. The results showed that UC-MSC infusions can reverse biochemical and histological abnormalities in the liver and improve metabolic disorders including hyperglycaemia and hyperlipidaemia. The observed hepatoprotection by UC-MSC therapy benefited from the modulation of lipid metabolism through promotion of *β*-oxidation and suppression of lipogenesis in the liver. Our results suggested that human UC-MSC infusions can serve as a novel and alternative therapy for patients with concomitant NAFLD and T2DM.

## 2. Materials and Methods

### 2.1. Isolation, Culture, and Characterization of Human UC-MSCs

Human umbilical cords were obtained from women giving birth in the First Medical Center of PLA General Hospital. All of the subjects provided informed consent. The experimental protocols were approved by the Medical Ethics Committee of PLA General Hospital. Human UC-MSCs were isolated and cultured by previously described methods [19]. The cultured UC-MSCs at passage 3 were digested and harvested to identify the immunophenotype using FITC-conjugated anti-CD45, anti-CD90, and anti-HLA-DR; PE-conjugated anti-CD105 and anti-CD73; and PerCP-conjugated anti-CD34 antibodies by flow cytometry analysis. All the antibodies used for surface marker analysis were purchased from BD Company. The potential of UC-MSCs to differentiate into osteoblasts and adipocytes was verified as previously described [18, 19].

### 2.2. Animal Models and Experimental Design

28-week-old male C57BL/6 db/db mice (*n* = 12) and age-matched C57BL/6 wide-type db/+ littermates (*n* = 6) were provided by the Animal Center of Peking University. All the animals were maintained on a standard diet and had free access to water in a temperature- and humidity-controlled environment under a 12 h light/dark cycle. After a two-week acclimation period, the db/db mice were randomly assigned to two groups: the UC-MSC-treated group (*n* = 6, referred as the db/db-MSC group) and the phosphate-buffered saline- (PBS-) treated group (*n* = 6, referred as the db/db-PBS group). For the experiment, db/db mice were infused with 1 × 10^6^ human UC-MSCs suspended in 0.2 ml PBS (for the db/db-MSC group) or with 0.2 ml PBS alone (for the db/db-PBS group) through the tail vein once a week for six weeks in succession. Meanwhile, the wild-type db/+ mice were used as a normal control (referred to as the db/+ group). The body weight and random blood glucose in each group were monitored weekly.

This study was approved by the Institutional Animal Care and Use Committee (IACUC) of PLA General Hospital. All the animal experiments complied with the standard ethical guidelines prescribed by the committee mentioned above.

### 2.3. Intraperitoneal Glucose Tolerance Test (IPGTT) and Intraperitoneal Insulin Tolerance Test (IPITT)

One week after the six-week treatment, an intraperitoneal glucose tolerance test (IPGTT) and an intraperitoneal insulin tolerance test (IPITT) were performed on the mice in the db/+, db/db-PBS, and db/db-MSC groups to evaluate the effect of UC-MSCs by previously described methods [17].

### 2.4. Blood and Tissue Collection

At the end of the experiment, mice were injected intraperitoneally with 1% pentobarbital sodium (50 mg/kg) anaesthesia. Blood was collected and centrifugated at 3000 rpm for 15 min to obtain serum for biochemical analyses. One-third of the fresh liver was rapidly excised and stored at −80°C for mRNA and protein assay. Then, the mice were perfused through the left ventricle with 10–15 ml PBS, followed by 10–15 ml of 4% paraformaldehyde. After the perfusion, the remaining liver was collected. One-half of the remaining tissue was fixed overnight in 4% paraformaldehyde and then embedded in paraffin to make cross sections of 3 *μ*m thickness for haematoxylin-eosin (HE) and Sirius Red staining. The HE staining was performed to assess hepatic steatosis and inflammation, while the Sirius Red staining was performed to assess fibrosis. Another half of the remaining tissue was incubated in 30% sucrose/PB overnight and then embedded (Tissue-Tek OCT Compound; Sakura Finetek, Torrance, CA) to make into frozen sections (5 *μ*m) for Oil Red O staining to detect hepatic lipid deposition and for immunofluorescence staining to examine the expression of fatty acid synthase (FASN).

### 2.5. Biochemical Analyses

The levels of alanine transaminase (ALT), aspartate aminotransferase (AST), total cholesterol (TC), triglyceride (TG), and low-density lipoprotein cholesterol (LDL-C) in the serum were measured using standard analytics (Beijing Kang Jia Hong Yuan Biological Technology Co. Ltd., China).

### 2.6. Histopathological Examinations and Immunofluorescence Analysis

HE, Oil Red O, and Sirius Red staining were carried out following standard procedures. For immunofluorescence staining, the frozen sections were incubated for 14 h at 4°C with antibody specific for FASN (1 : 1000, rabbit, Abcam). The slides were then incubated for 2 h at room temperature with the donkey anti-rabbit secondary antibody (1 : 500, Alexa-594, Invitrogen). The nuclei were visualized with 4′,6-diamidino-2-phenylindole (DAPI) (Sigma). The HE, Sirius Red, and Oil Red O staining sections were photographed by a light microscope (Leica TCS SP2, Germany) while the immunofluorescence staining sections were captured by a laser scanning confocal microscope (Leica, Wetzlar, Germany). Evaluation of the extent of NAFLD was performed using the NAFLD activity score (NAS) [20].

### 2.7. Quantitative Real-Time Reverse Transcriptase Polymerase Chain Reaction (qRT-PCR)

Total RNA was extracted from liver tissue, and single-stranded cDNA were synthesized with a reverse transcription kit (Thermo Scientific, CA, USA). Real-time quantitative polymerase chain reaction (PCR) analysis was conducted with a SYBR Green PCR master mix (Applied Biosystems) on ABI Prism thermal cycler model StepOnePlus (Applied Biosystems, CA, USA). The thermal cycling program was 95°C for 5 min, followed by 95°C for 15 s, 60°C for 30 s, and 72°C for 30 s for 40 cycles. Melting curve analysis was performed to ensure the specificity of primers. GAPDH was used as a reference gene in each sample. The analysis for target gene expression was performed using the relative quantification comparative CT method. The primer sequences used in the qRT-PCR were shown in Supplementary [Supplementary-material supplementary-material-1].

### 2.8. Western Blot

Mouse liver tissue was lysed in lysis buffer, and protein concentration was determined by a BCA Protein Assay Kit (Beyotime, China). Aliquots containing 30 *μ*g of protein were used for western blot analyses, and the experiment was performed following the standard procedure. The primary antibodies were HNF4*α* (1 : 1000, rabbit, Abcam), CES2 (1 : 1000, rabbit, ZenBio), ACC (1 : 1000, rabbit, CST), and *β*-actin (1 : 2000, mouse, ZSGB-Bio). The secondary antibodies were goat anti-rabbit (1 : 3000) and rabbit anti-mouse (1 : 2500) IgG horse radish peroxidase (HRP, ZSGB-Bio). *β*-Actin was loaded as an internal control, and the proteins were quantified by the use of ImageJ software (NIH, Bethesda, MD, USA).

### 2.9. Statistical Analysis

The results were expressed as means ± SD (standard deviation). The statistical analysis was carried out using the SPSS 19.0 software (SPSS Inc., IBM, USA). Differences between means were assessed using *t*-tests (two samples) or one-way ANOVA (three or more samples). For all analyses, the statistical significance level was set at *p* < 0.05.

## 3. Results

### 3.1. Characterization of Human UC-MSCs

**Figure 1:**
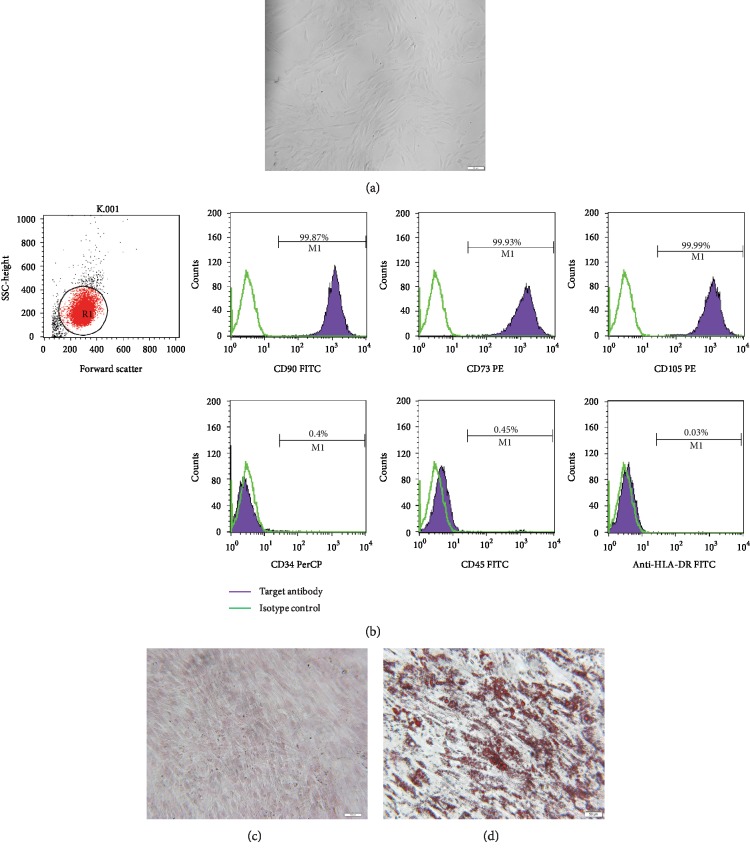
Identification of human UC-MSCs. (a) Morphological features. The MSCs appeared spindle-like and fibroblastoid-shaped. Scale bar = 100 *μ*m. (b) Flow cytometric analysis of the expression of cell surface markers related to human UC-MSCs. The expression of each antigen was presented with the corresponding isotype control. (c) Alizarin Red S staining of cultured osteogenic human UC-MSCs. Scale bar = 100 *μ*m. (d) Oil Red O staining of cultured adipogenic human UC-MSCs. Scale bar = 50 *μ*m.

The cultured human UC-MSCs have a bipolar spindle-like and fibroblastoid-shaped morphology (Figure 1(a)). To further identify the adherent cells, immunophenotypic features and multilineage differentiation potential were examined. As presented in Figure 1(b), the cells expressed surface marker characteristic of UC-MSCs, including CD90, CD73, and CD105, while negative surface markers of UC-MSCs, including CD34, CD45, and HLA-DR, were not expressed. Moreover, UC-MSCs exhibited potential to differentiate into osteoblasts (Figure 1(c)) and adipocytes (Figure 1(d)).

### 3.2. Human UC-MSC Infusions Improved Glucose Homeostasis in db/db Mice

**Figure 2:**
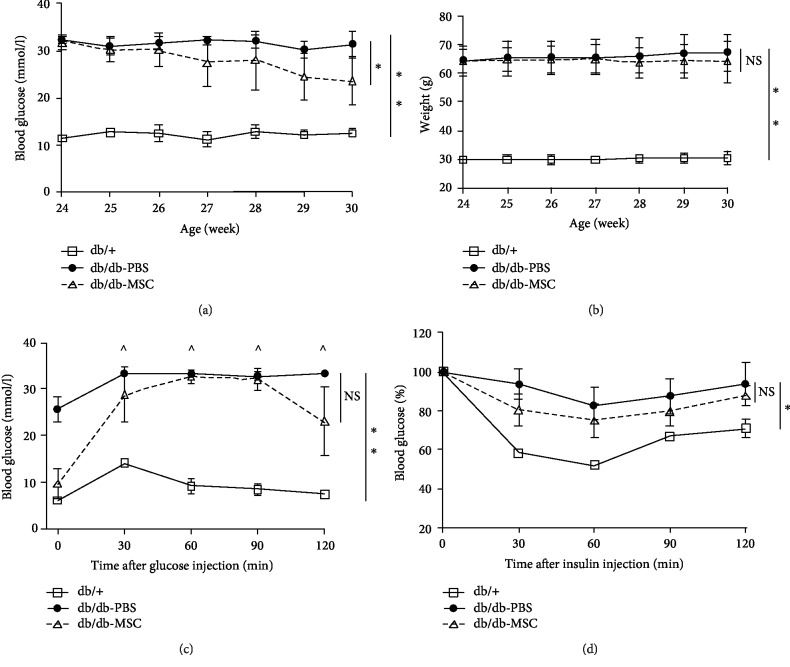
Human UC-MSC infusions improved glucose homeostasis. db/db mice were infused with human UC-MSCs or PBS once a week for six weeks. Blood glucose levels (a) and body weight (b) of the indicated groups were monitored once a week during the whole experiment period. (c) IPGTT was performed on the indicated groups to assess glucose tolerance. ^ indicates that the blood glucose level exceeded the maximum (33.3 mmol/l) of the glucometer. (d) IPITT was performed on the indicated groups to assess insulin tolerance, and the results were presented relative to the initial blood glucose levels. All the results are expressed as the means ± SD. *n* = 6 mice per group. ^∗^*p* < 0.05; ^∗∗^*p* < 0.01. ns: not significant; IPGTT: intraperitoneal glucose tolerance tests; IPITT: intraperitoneal insulin tolerance tests.

Due to the dysfunction of the leptin receptor, db/db mice spontaneously develop hyperphagia-induced obesity, insulin resistance, and T2DM. At baseline, the random blood glucose level and the body weight were significantly elevated in the db/db mice compared to those in the wild-type db/+ mice (31.0 ± 2.0 mmol/l vs. 12.7 ± 0.6 mmol/l, *p* < 0.05; 65.5 ± 6.0 g vs. 30.0 ± 1.8 g, *p* < 0.05). After UC-MSC treatment, the db/db mice presented a dramatic fall in the blood glucose level, while the PBS-treated mice remained persistent hyperglycaemic (21.9 ± 4.3 mmol/l vs. 30.8 ± 3.7 mmol/l, *p* < 0.05; Figure 2(a)). Meanwhile, UC-MSCs slightly reduced the body weight of db/db mice, but the change was not statistically significant (62.8 ± 8.5 g vs. 68.7 ± 6.8 g, *p* = 0.32; Figure 2(b)). Since T2DM is featured in glucose intolerance and insulin resistance, we carried out glucose tolerance and insulin tolerance tests. During the IPGTT test (Figure 2(c)), the fasting blood glucose in the db/db-MSC group was markedly lower than that in the db/db-PBS group (9.5 ± 3.2 mmol/l vs. 25.6 ± 2.6 mmol/l, *p* < 0.01). Starting from 30 minutes, the blood glucose in the db/db-PBS group remained “high” (marked with “^” in the figure), exceeding the maximum value (33.3 mmol/l) that could be detected by the glucometer, and those data were uniformly recorded as 33.3 mmol/l. Meanwhile, the db/db-MSC group showed a significant decrease in the blood glucose level at 120 min, indicating a notable improvement in the glucose metabolism. During the IPITT test, the MSC-treated mice were more sensitive to insulin than the diabetic control (Figure 2(d)). Taken together, the results suggested that UC-MSC infusions improved glucose homeostasis in db/db mice.

### 3.3. Human UC-MSC Infusions Relieved Hepatic Functional Injury and Improved Lipid Profiles in db/db Mice

**Figure 3:**
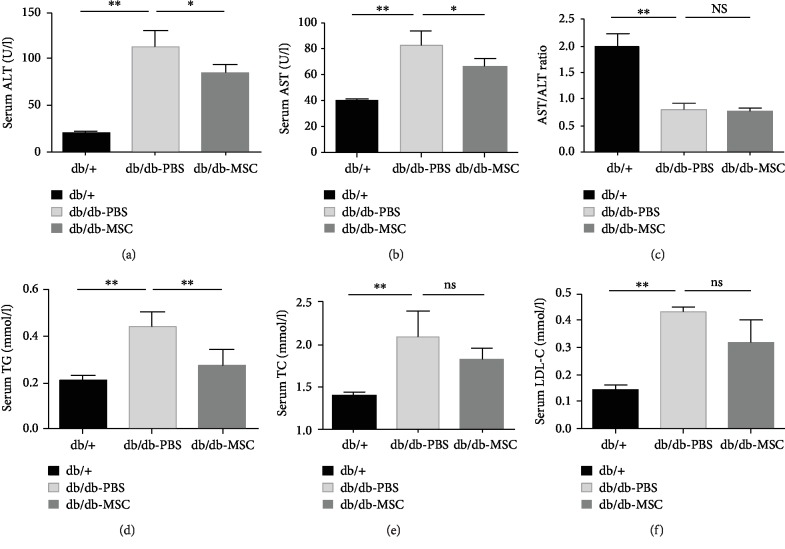
Human UC-MSC infusions relieved functional hepatic injury and improved lipid profiles. db/db mice were infused with human UC-MSCs or PBS once a week for six weeks. One week after the infusions, serum concentrations of ALT (a), AST (b), AST/ALT ratio (c), TG (d), TC (e), and LDL-C (f) in the indicated groups were measured. All data are represented as means ± SD. *n* = 6 mice per group. ^∗^*p* < 0.05; ^∗∗^*p* < 0.01. ns: not significant.

Functional hepatic injury was evaluated by determining the serum ALT and AST levels. The results displayed that the ALT and AST levels were markedly increased in the db/db-PBS group compared to that in the db/+ group (113.7 ± 17.3 U/l vs. 20.3 ± 17.3 U/l, *p* < 0.01; 82.6 ± 11.8 U/l vs. 39.8 ± 2.0 U/l, *p* < 0.01), indicating the impaired liver function under NAFLD and T2DM conditions, while infusions of UC-MSCs significantly decreased the elevated levels of ALT and AST in db/db mice (87.1±8.1 U/l vs. 113.7±17.3 U/l, *p* < 0.05; 66.1 ± 11.8 U/l vs. 82.6 ± 11.8 U/l, *p* < 0.05), suggesting that the hepatic functional injury was alleviated by UC-MSC treatment (Figures 3(a) and 3(b)). Moreover, the AST to ALT (AST/ALT) ratio, a useful index to distinguish nonalcoholic steatohepatitis from alcoholic liver disease and a significant predictor of long-term complications such as fibrosis and cirrhosis [21, 22], was evaluated here. The results (Figure 3(c)) showed that both the db/db-PBS group and the db/db-MSC group presented an AST/ALT ratio less than 1.0, with a decline from 0.83 ± 0.13 in the db/db-PBS group to 0.71 ± 0.09 in the db/db-MSC group. In addition, the progression of NAFLD was assessed by detecting serum TG, TC, and LDL-C levels. As shown in Figures 3(d)–3(f), the high levels of serum TG, TC, and LDL-C were lowered by UC-MSCs, among which the decrease of TG was the most obvious, with statistical significance (db/db-MSC group vs. db/db-PBS group: 0.28 ± 0.07 mmol/l vs. 0.44 ± 0.06 mmol/l, *p* < 0.01). These results demonstrated that UC-MSC infusions improved hepatic function and attenuated hyperlipidaemia in db/db mice.

### 3.4. Human UC-MSC Infusions Ameliorated Hepatic Steatosis in db/db Mice

**Figure 4:**
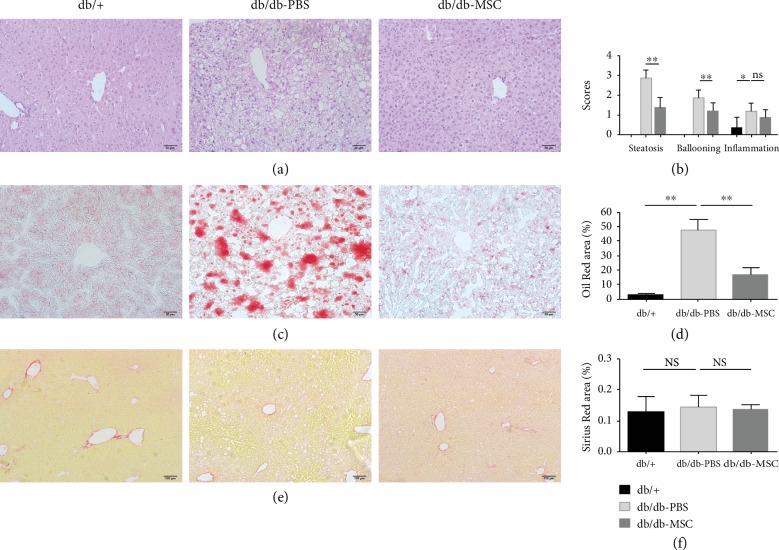
Human UC-MSC infusions ameliorated hepatic steatosis. (a) Representative images of HE-stained liver sections of the indicated groups. Scale bar = 50 *μ*m. (b) Steatosis, ballooning, and inflammation in the liver of the indicated groups were scored. (c) Representative images of Oil Red O-stained liver sections of the indicated groups. Scale bar = 50 *μ*m. (e) Representative images of Sirius Red-stained liver sections of the indicated groups, scale bar = 100 *μ*m. Quantitative analysis of (d) the Oil Red O-positive area and (f) the Sirius Red-positive area in the respective groups. *n* = 5 sections per group. All data are represented as means ± SD. ^∗^*p* < 0.05; ^∗∗^*p* < 0.01. ns: not significant.

To assess whether UC-MSCs could alleviate hepatic morphological changes in db/db mice, we performed histopathological examinations on liver tissues. HE staining showed normal hepatic lobules with regularly arranged hepatocytes and small lipid droplets in the db/+ group. The db/db-PBS group presented obvious steatosis indicated by enlarged hepatocytes containing lipid droplets of various sizes, with a distinct hepatocellular ballooning and more than 90% parenchymal steatosis. Moreover, occasional inflammation was observed in the liver of the db/db-PBS group. In comparison, the db/db-MSC group displayed smaller-sized hepatocytes with fewer intracellular lipid droplets, alleviated ballooning injury, and less than 40% parenchymal steatosis. There were no changes in the inflammation in the liver of the db/db-MSC group compared to that in the db/db-PBS group (Figures 4(a) and 4(b)). Additionally, the Oil Red O-positive area exhibited an obvious enlargement in PBS-treated mice and an evident reduction in the MSC-treated mice, which indicated the decrease of lipid deposition in the liver after UC-MSC treatment (Figures 4(c) and 4(d)). However, no obviously increased fibrosis was detected in the PBS-treated mice or MSC-treated mice by Sirius Red staining (Figures 4(e) and 4(f)). Together, these results demonstrated that UC-MSC infusions ameliorated hepatic steatosis and reduced lipid accumulation in db/db mice.

### 3.5. Human UC-MSC Infusions Regulated Lipid Metabolism by Promoting *β*-Oxidation and Suppressing Lipogenesis

**Figure 5:**
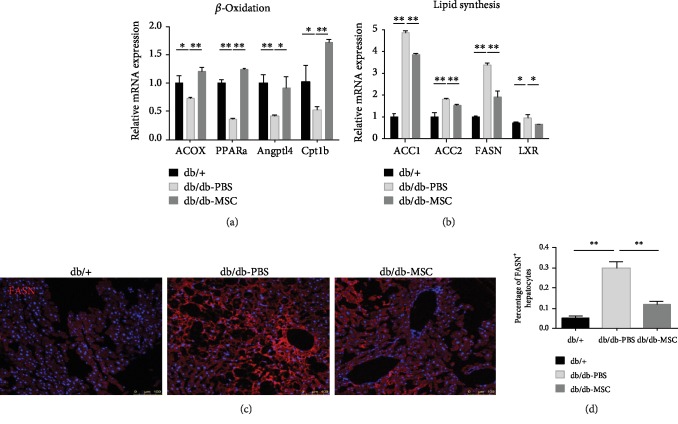
Human UC-MSC infusions promoted *β*-oxidation and suppressed lipogenesis. Transcript levels of genes related to *β*-oxidation (a) and lipogenesis (b) in the livers of the indicated groups assessed by qRT-PCR. Each experiment was repeated 3 times. (c) Immunofluorescence staining for FASN-positive hepatocytes (bright red fluorescence) in representative liver sections of the indicated groups (*n* = 5 sections per group). (d) The proportion of FASN-positive hepatocytes was quantified. All the data are expressed as means ± SD. ^∗^*p* < 0.05; ^∗∗^*p* < 0.01.

Lipid metabolism is an important pathway involved in the development of NAFLD, and imbalance between lipolysis and lipogenesis contributes to the lipid accumulation in the hepatocytes [23, 24]. To explore the underlying mechanism by which UC-MSCs improved lipid profiles and attenuated hepatic steatosis, we assessed the transcript levels of genes related to fatty acid *β*-oxidation (PPAR*α* and its target genes ACOX1, Angplt4, and Cpt1b) and lipid synthesis (LXR, ACC1, ACC2, and FASN) in the livers of the indicated groups by qRT-PCR. The results showed that compared to the db/+ mice, the db/db mice had remarkably decreased expression levels of *β*-oxidation-related genes and increased expression levels of lipid synthesis-related genes. After UC-MSC infusions, the oxidation-related genes were significantly upregulated and lipogenesis-related genes were markedly downregulated (Figures 5(a) and 5(b)). To further investigate the effect of UC-MSCs on lipid metabolism, we applied immunofluorescence staining on liver sections to detect FASN, one of the step-limiting enzymes for de novo lipogenesis. The results showed that the proportion of FASN-positive (FASN+) hepatocytes (bright red fluorescence) presented a marked rise in the PBS-treated mice and a significant decline in MSC-treated mice (db/db-MSC group vs. db/db-PBS group: 11.8 ± 1.7% vs. 29.9 ± 3.2%, *p* < 0.01; Figures 5(c) and 5(d)), which suggested that UC-MSC treatment suppressed the activity of lipogenesis. Taken together, these results indicated that UC-MSC infusions can regulate lipid metabolism by promoting fatty acid *β*-oxidation and suppressing lipogenesis, which accounted for the amelioration of NAFLD.

### 3.6. Upregulation of the HNF4*α*-CES2 Pathway Was Involved in the Curative Effect of UC-MSCs on NAFLD

**Figure 6:**
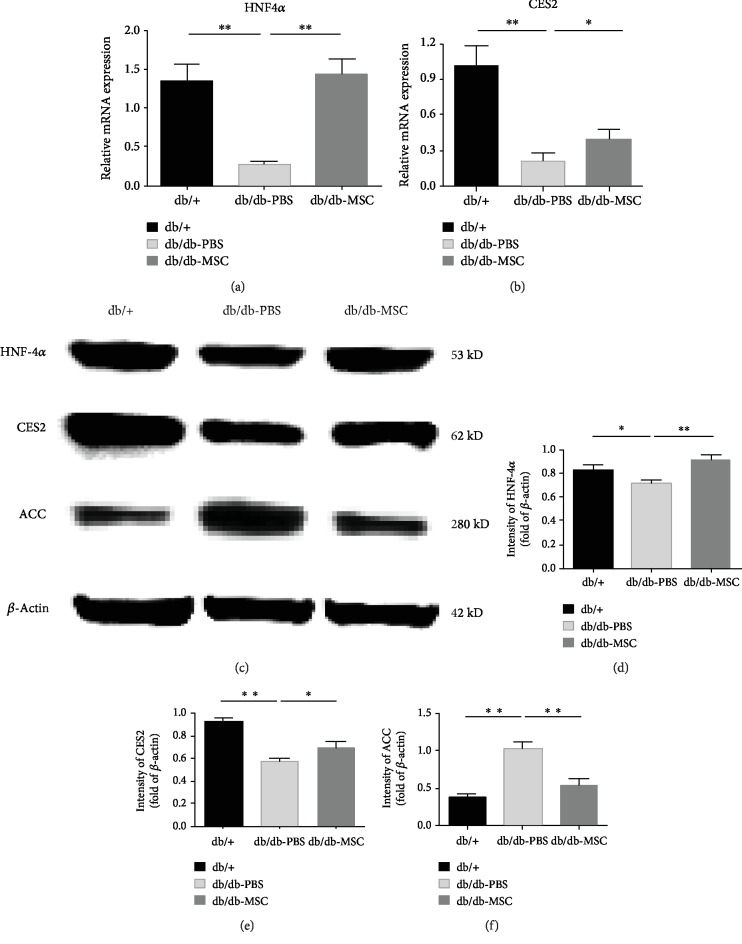
Human UC-MSC infusions upregulated the HNF4*α* and CES2 expressions. Transcript levels of HNF4*α* (a) and CES2 (b) in the livers of the indicated groups assessed by qRT-PCR. (c) Protein levels of HNF4*α*, CES2, and ACC assessed by western blot (*β*-actin served as the loading control). (d–f) Statistical analysis of HNF4*α*, CES2, and ACC protein levels. Each experiment was repeated three times, and typical pictures were shown. Data are expressed as means ± SD. ^∗^*p* < 0.05; ^∗∗^*p* < 0.01.

Using experimental animal models and clinical samples, recent studies have demonstrated that CES2 and its upstream regulator HNF4*α* control the expression of numerous genes involved in lipid metabolism and are essential for maintaining normal lipid homeostasis in the liver [25, 26]. CES2 possesses triglyceride and diacylglycerol lipase activities, and knockdown of HNF4*α* or CES2 reduces fatty acid oxidation and increases de novo lipogenesis [27, 28]. Accordingly, we speculated that the HNF4*α*-CES2 pathway may be involved in the observed therapeutic effect of UC-MSCs on NAFLD, and then we measured the activity of the pathway among the experimental groups by qRT-PCR and western blot. The results showed that at both the mRNA level (Figures 6(a) and 6(b)) and the protein level (Figures 6(c)–6(e)), the expressions of HNF4*α* and CES2 dramatically decreased under NAFLD and diabetes conditions and that UC-MSC infusions significantly augmented them. As a result, increased CES2 could hydrolyse TG to release FFA and inhibit LXR activity, which can stimulate free fatty acid (FFA) synthesis. For instance, acetyl-CoA carboxylase (ACC), a rate-limiting enzyme of fatty acid synthesis in the liver, was suppressed by UC-MSC treatment (Figures 6(c) and 6(f)). Meanwhile, increased CES2 could also enhance PPAR*α* activity that can promote fatty acid *β*-oxidation by activating its target genes, including Cpt1b and Angplt4 which showed an increase after UC-MSC infusions (Figure 5(a)). Taken together, these results suggested that the therapeutic effect of UC-MSCs on NAFLD was associated with the upregulation of the HNF4*α*-CES2 pathway in the liver.

## 4. Discussion

NAFLD is becoming a globally epidemic disease in children and adults, which is associated with metabolic disorders, such as obesity, T2DM, and hyperlipidaemia. The coexistence of NAFLD and T2DM often results in worse metabolic profile and higher risks of cardiovascular events, advanced fibrosis or cirrhosis, and hepatocellular carcinoma [29]. However, there are no approved pharmacological agents specifically designed for NAFLD currently, and in addition to the lifestyle modifications including dietary changes and increased physical activity, other therapeutic options for patients with NAFLD are limited [30].

UC-MSCs have attractive advantages such as a noninvasive collection procedure, lower immunogenicity, faster self-renewal ability, and fewer ethical constraints compared with other stem cells. UC-MSC transplantation has made encouraging progress in treating dozens of socially significant diseases, such as acute myocardial infarction and diabetes mellitus types I and II, and autoimmune and neurodegenerative diseases [9, 31, 32]. In this study, we examined the possible therapeutic effect of UC-MSCs on NAFLD in type 2 diabetic db/db mice. The results showed that UC-MSC treatment alleviated hepatic functional injury, attenuated hepatic steatosis, and reduced hepatic lipid accumulation, as evidenced by markedly decreased serum levels of ALT and AST, lowered AST/ALT ratio, and alleviated histological lesion of liver tissue. Meanwhile, UC-MSC infusions ameliorated glucose and lipid metabolic disorder, demonstrated by significantly improved glucose tolerance in IPGTT and lowered serum levels of TG, TC, and LDL-C. However, in this study, the efficacy of UC-MSCs was evaluated one week after the infusions; further research is needed to observe the duration of efficacy of UC-MSC treatment. Additionally, our results showed that the body weight was not reduced markedly and the insulin sensitivity was not improved significantly in the MSC-treated mice. Whether increasing the total number of injections and number of injected cells could provide a better therapeutic effect needs further exploration.

The AST/ALT ratio is an important indicator for etiological analysis of liver diseases, as the ratio is usually less than 1.0 in NAFLD and greater than 2.0 in alcoholic hepatitis [21]. Moreover, since the increase in transaminases is generally mild in NAFLD and when they are elevated, the level of ALT is usually higher than that of AST; the AST/ALT ratio is more characteristic than the transaminase levels and holds potential in evaluating the progression of fibrosis in the liver [22, 33]. It is believed that the AST/ALT ratio greater than 1.0 is an independent predictor of advanced liver fibrosis in NASH and the ratio increases as the fibrosis progresses [34–37]. In the present study, both the db/db-PBS group and the db/db-MSC group showed an AST/ALT ratio below 1.0, consistent with the results of the Sirius Red staining that exhibited not obvious fibrosis in the indicated groups. It is necessary to point out that similar to fibrosis, liver inflammation was also not evident in the db/db mice, indicating that the normal diet-fed db/db mice were still in the early stage of NAFLD and they did not develop NASH, which is a severe form of NAFLD. Interestingly, it is reported that when fed methionine-choline-deficient (MCD) diet for several weeks, db/db mice can develop NASH characterized by pronounced histological inflammation and fibrosis in the liver [38, 39]. Thus, further research is needed to study the efficacy of UC-MSCs on MCD diet-fed db/db mice, enriching the experimental basis of UC-MSC therapy for NAFLD.

Hepatic steatosis results from lipid metabolism disorders characterized by an imbalance between lipolysis and lipogenesis. It has been demonstrated in different disease models that MSCs possess the ability to modulate lipid metabolism [40, 41]. In the present study, we showed that the expression levels of *β*-oxidation-related genes were increased and those of lipogenesis-related genes were decreased upon UC-MSC treatment, indicating that the UC-MSCs could restore the alterations in lipid metabolism in NAFLD, which underlay the attenuated hyperlipidaemia in db/db mice. HNF4*α* is a member of the nuclear receptor superfamily and is especially expressed in the liver, kidney, small intestine, pancreas, and stomach [42, 43]. This nuclear receptor is a pivotal regulator controlling lipid or glucose metabolism in the liver. Liver-specific conditional knockout of HNF4*α* in adult mice led to severe steatosis associated with disruption of VLDL secretion and misregulation of ApoB and MTTP expression [27, 44]. CES2 is a highly abundant esterase in both the liver and the intestine, possessing triglyceride and diacylglycerol lipase activities. In primary human hepatocytes, CES2 knockdown impaired glucose storage and lipid oxidation [28]. It has been demonstrated that HNF4*α* regulates CES2 expression by transactivating the CES2 gene promoter [25]. In the present study, we demonstrated that the expression levels of HNF4*α* and CES2, which declined dramatically in the PBS-treated db/db mice, were restored by UC-MSC infusions. It can be postulated that the increased CES2 could promote FFA release and the released FFAs may function as a signalling molecule by activating PPAR*α*, a nuclear receptor that regulates fatty acid oxidation. Meanwhile, increased CES2 could also inhibit lipogenic genes and alleviate endoplasmic reticulum (ER) stress in the liver [45]. Therefore, it can be inferred that the therapeutic effect of UC-MSCs on NAFLD in db/db mice was associated with the upregulation of the HNF4*α*-CES2 pathway. Further studies utilizing liver-specific HNF4*α* or CES2 knockout mice are needed to confirm the essential role of the HNF4*α*-CES2 pathway in the treatment of NAFLD by UC-MSCs.

## 5. Conclusions

In summary, we demonstrated that human UC-MSC infusions significantly attenuated hyperglycaemia, improved lipid metabolism, and ameliorated NAFLD in db/db mice, which may be associated with the upregulation of the HNF4*α*-CES2 pathway that promoted *β*-oxidation and inhibited lipogenesis in the liver. UC-MSC-based therapy may become an ideal option for patients with concomitant NAFLD and T2DM.

## Data Availability

The data used to support the findings of this study are available from the first author upon request.

## Supplementary Materials

Supplementary Materials Supplementary Table 1: the primer sequences of target genes detected by RT-PCR.
