# Supplementary material for: Human Umbilical Cord-Derived Mesenchymal Stem Cell Therapy Ameliorates Nonalcoholic Fatty Liver Disease in Obese Type 2 Diabetic Mice
Source: Stem Cells Int. 2019 Nov 3;2019:8628027. doi: 10.1155/2019/8628027 (PMC6875176; doi:10.1155/2019/8628027)
Supplement: Supplementary Materials — Supplementary Table 1: the primer sequences of target genes detected by RT-PCR. [file 8628027.f1.docx]

**Supplementary information**

Supplementary table 1 showed the primer sequences of target genes detected by the RT-PCR.

Supplementary Table 1 The primers used to perform RT-PCR.

| **Gene Forward Sequence Reverse Sequence** |
| --- |
| GAPDH ACTCCCACTCTTCCACCTTC TCTTGCTCAGTGTCCTTGC  ACOX1 TCCATGTTTATCCCTACCTTGCT CTCGAAGATGAGTTCCGTGGC  PPARα TGCAGCCTCAGCCAAGTTGAA AGCCACAAACGTCAGTTCACA  Angptl4 CTTCCACTCTATCCCACGGC TAGCGGCCCTTCCATGTTTT  Cpt1b GCTACACGGAGACAGGACAC ACGAGTTCTCGATGGCTTCC  ACC1 GGGAACATCCCCACGCTAAA CATGCGTTGACAAGGTGGTG  ACC2 TTCCCCAGCCAGCAGATAG TGGGCTTGCTGAAAATGGTG FASN GGCCCCTCTGTTAATTGGCT GGATCTCAGGGTTGGGGTTG  LXR GACAGTTTTGGTAGAGGGACA GTAGGCTCTGCTGACTCCAA HNF4α CGTGTGTGACCCCATAGGAC GTTCTTCCTCACGCTCCTCC  CES2 CCTGTAGGACCACTGCGATT ACATAGGGAAGGAAGACAGCATC |

ACOX1, Acyl-coenzyme A oxidase 1; PPARα, Peroxisome proliferator-activated receptor α; Angplt4, Angiopoietin like protein 4; Cpt1b, Carnitine palmitoyltransferase 1b; ACC1/2, Acetyl-CoA carboxylase 1/2; FASN, Fatty acid synthase; LXR, liver X receptor; HNF4α, Hepatocyte nuclear factor-4; CES2, Carboxylesterase 2.
